# Relating MR relaxation times of *ex vivo* meniscus to tissue degeneration through comparison with histopathology

**DOI:** 10.1016/j.ocarto.2020.100061

**Published:** 2020-04-03

**Authors:** Emma Einarsson, Jonas Svensson, Elin Folkesson, Iida Kestilä, Jon Tjörnstrand, Pernilla Peterson, Mikko A.J. Finnilä, H. Velocity Hughes, Aleksandra Turkiewicz, Simo Saarakkala, Martin Englund

**Affiliations:** a Medical Radiation Physics, Department of Translational Medicine, Lund University, Malmö, Sweden; b Clinical Epidemiology Unit, Orthopedics, Department of Clinical Sciences Lund, Lund University, Lund, Sweden; c Medical Imaging and Physiology, Skåne University Hospital, Lund, Sweden; d Molecular Skeletal Biology and Rheumatology, Department of Clinical Sciences Lund, Lund University, Lund, Sweden; e Research Unit of Medical Imaging, Physics and Technology, University of Oulu, Oulu, Finland; f Orthopedics, Department of Clinical Sciences Lund, Lund University, Skåne University Hospital, Lund, Sweden; g Department of Diagnostic Radiology, Oulu University Hospital, Oulu, Finland

**Keywords:** MRI, Relaxation times, Histology, Ex vivo, Meniscus, Osteoarthritis

## Abstract

**Background:**

Quantitative magnetic resonance imaging (MRI), e.g. relaxation parameter mapping, may be sensitive to structural and compositional tissue changes, and could potentially be used to non-invasively detect and monitor early meniscus degeneration related to knee osteoarthritis.

**Objective:**

To investigate MR relaxation times as potential biomarkers for meniscus degeneration through comparisons with histopathology.

**Methods:**

We measured MR relaxation parameters in the posterior horn of 40 menisci (medial and lateral) at a wide range of degenerative stages. T1, T2 and T2^∗^ were mapped using standard and ultrashort echo time sequences at 9.4 T and compared to gold standard histology using Pauli's histopathological scoring system, including assessment of surface integrity, collagen organization, cellularity and Safranin-O staining.

**Results:**

All three relaxation times increased with total Pauli score (mean difference per score (95% CI) for T2^∗^: 0.62 (0.37, 0.86), T2: 0.83 (0.53, 1.1) and T1: 24.7 (16.5, 32.8) ms/score). Clear associations were seen with scores of surface integrity (mean difference per score for T2^∗^: 3.0 (1.8, 4.2), T2: 4.0 (2.5, 5.5) and T1: 116 (75.6, 156) ms/score) and collagen organization (mean difference between highest and lowest score for T2^∗^: 5.3 (1.6, 8.9), T2: 6.1 (1.7, 11) and T1: 204 (75.9, 332) ms). The results were less clear for the remaining histopathological measures.

**Conclusions:**

MR relaxation times T1, T2 and T2^∗^ of *ex vivo* human menisci are associated with histologically verified degenerative processes, in particular related to surface integrity and collagen organization. If confirmed *in vivo,* MR relaxation times may thus be potential biomarkers for meniscus degeneration.

## 1 Introduction

It has been reported that meniscus damage following tissue degeneration is associated with the development of knee osteoarthritis (OA) [1,2]. To further investigate the role of the meniscus in OA, we need a non-invasive method to detect and monitor early meniscus degeneration. Standard clinical magnetic resonance imaging (MRI) and semi-quantitative assessment allow for detection of meniscal tears and destruction, but are relatively insensitive to early intra meniscal degeneration of the tissue [3]. Quantitative MRI, however, e.g. mapping of MR relaxation times, may be useful for assessment of tissue composition and degenerative changes before the appearance of macroscopic damage. Ultrashort echo time (UTE) sequences are advantageous in short T2 tissues, such as the meniscus [4], and mapping of T2^∗^ using such sequences may thus be of particular interest.

We recently reported that T2^∗^, T2 and T1 relaxation times are longer in the posterior horn of *ex vivo* medial menisci from medial compartment knee OA patients compared to contralateral and reference menisci [5]. These results suggest that changes in MR relaxation parameters may be indicative of OA-related meniscus degeneration. However, a comparison between groups of menisci from knees with severe OA and without known OA does not reveal if relaxation times also reflect more subtle differences in disease severity. Further, such a comparison does not have the potential of investigating the influence of different degenerative processes.

Before proceeding into longitudinal *in vivo* studies it would therefore be advantageous to compare relaxation times to a gold standard method for evaluation of meniscus degeneration, such as histopathological scoring, which have shown promising results in earlier studies [[6], [7], [8]]. For instance, Nebelung et al. measured MR relaxation times in lateral menisci from patients undergoing total knee replacement and reported that higher grades of degeneration were associated with longer relaxation times [6] and Eijgenraam et al. recently reported that *in vivo* meniscus T2 is associated with histopathological score in patients with end-stage knee OA [8].

To further investigate if an increase in MR relaxation times reflect degeneration severity, examination of both healthy and diseased knee joints and both medial and lateral menisci, would be preferable to ensure a wide range of degenerative stages. Examination of histopathological scores for separate features of degeneration may also shed some light on which specific processes affect the relaxation times.

We have previously reported MR relaxation times and histopathological analysis in two separate studies, comparing groups of healthy and diseased *ex vivo* human meniscus samples [5,9]. Using this material, that covers a wide range of degenerative stages, we now aim to relate MR relaxation times, T2^∗^, T2 and T1 to meniscus degeneration evaluated through gold standard histopathological scoring to investigate the potential of relaxation times as biomarkers for progressing meniscus degeneration.

## 2 Methods

### 2.1 Tissue samples

We used human menisci from the MENIX biobank at Skåne University Hospital, Lund, Sweden. A total of 40 menisci were sampled from 10 medial compartment knee OA patients undergoing total knee replacement (TKR) and 10 deceased donors without known knee OA. Both medial and lateral menisci from the subjects were evaluated. Mean age (range) of the OA patients (5 men and 5 women) was 63 (50–75) years and of the donors (5 men and 5 women) 51 (18–77) years. The lateral compartments of the patients’ knees were visually unaffected by OA with Outerbridge grade 0. The study was approved by the local ethics committee (Dnr 2015–39 and 2016–865) and informed consent was collected directly from the patients or, in the case of the deceased donors, either via the donor register or from close relatives.

**Fig. 1:**
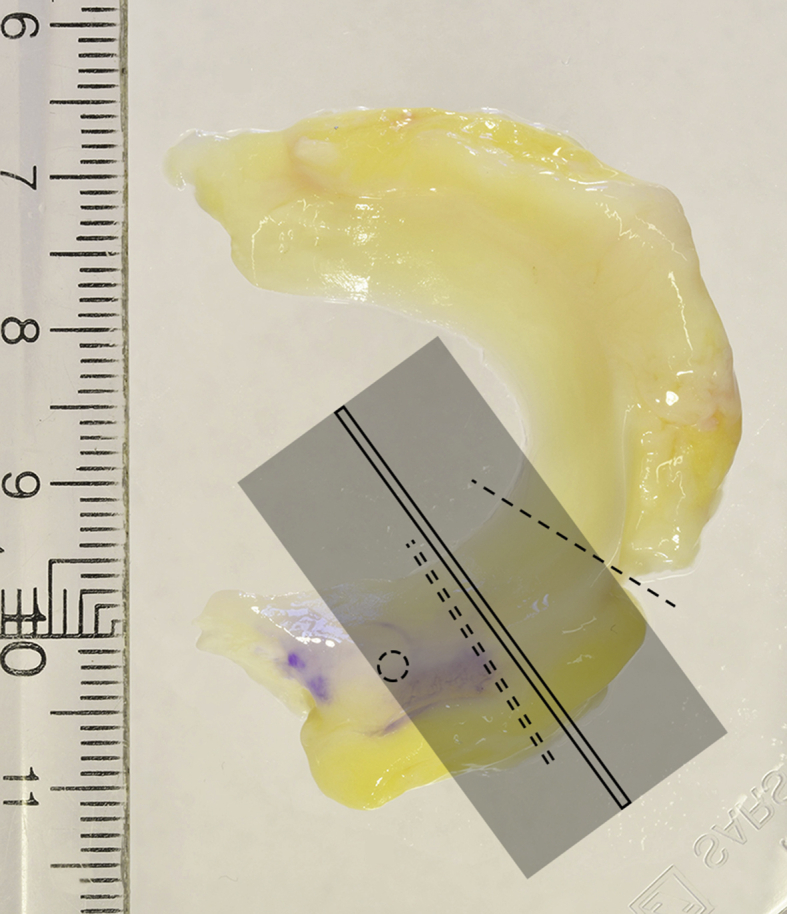
Schematic image showing the part of the meniscus sample that was used for the various measurements. The dashed line (single) indicates where the posterior horn was separated from the body. The circle shows where a hole was punched to enable stable positioning of the sample during MRI. The shaded field represents the volume covered by the MR image slices with the chosen slice for calculation of relaxation time marked by a solid rectangle. The dashed line (double) represents the approximate position of the slice cut out for the histopathologic analysis. The exact positions of the image slice used for relaxation time calculation and the histopathological analysis could vary somewhat but were generally in close vicinity to each other.

The samples were stored at −80 °C (mean time 358 ± 196 days) between collection and measurements. The posterior horn of each meniscus was cut out from the thawed meniscus and fixated inside a 50-ml plastic tube filled with phosphate buffered saline (PBS) in which it was imaged (Fig. 1).

### 2.2 MRI

MR relaxation times were measured using a 9.4 T preclinical scanner (Magnet: Agilent, Santa Clara, USA, Electronics: Bruker BioSpec AVIII, Bruker, Ettlingen, Germany) according to a protocol previously described [5]. Care was taken to position the meniscus in an orientation towards the main magnetic field similar to an *in vivo* measurement, i.e. with the main direction of the collagen fibres perpendicular to B_0_. A UTE sequence with eight echo times (TEs) of 0.5–12 ms was used to map T2^∗^. T2 was mapped using a standard Rapid Acquisition with Relaxation Enhancement (RARE) sequence with seven TEs of 4.7–17 ms and T1 was mapped using a RARE Variable Repetition Time (RAREVTR) sequence with six repetition times (TRs) of 200–6000 ms. For details on the image acquisition, see Table 1. T2 and T2^∗^ maps were calculated through voxel-by-voxel mono-exponential fitting of the signal at different TEs. T1 maps were generated directly at the scanner using the built-in T1-mapping protocol.

**Table 1:** Imaging parameters for the mapping of T2^∗^, T2 and T1.

Parameter	T2^∗^	T2	T1
Sequence	2D single echo UTE	2D single echo RARE	2D RAREVTR
TE	0.5, 1, 2, 4, 6, 8, 10 and 12 ms	4.7, 7, 9, 11, 13, 15 and 17 ms	6 ms
TR	17.5 ms	1500 ms	200, 400, 800, 1500, 3000 and 6000 ms
Flip angle	10°	90°	90°
BW	391 Hz/pixel	385 Hz/pixel	637 Hz/pixel
FOV	58 × 58 mm^2^	35 × 35 mm^2^	58 × 58 mm^2^
Number of slices	7	7 or 12	7
Voxel size	0.23 × 0.23 × 1 mm^3^	0.14 × 0.14 × 1 mm^3^	0.45 × 0.45 × 1 mm^3^
Acquisition time	8 × 3 min 17 s	7 × 2 min 24 s	9 min 43 s

Mean values of T2^∗^, T2 and T1 were calculated within a region of interest (ROI), drawn manually by one observer (EE) within one slice of each meniscus, covering the entire meniscus cross section except for a small margin towards the surrounding PBS to avoid partial volume effects. The slice was chosen centrally in the sample while avoiding artifacts from small air bubbles and calcifications. Artifact areas adjacent to air bubbles or calcifications, or where the tissue was clearly not intact (e.g. torn or fringed), were excluded when drawing the ROI.

After MRI, the meniscus sample was fixated in formalin before subsequent preparation for the histopathological analysis.

### 2.3 Histopathology

Histological preparation of the tissue was conducted following a standard assay as previously described by Kestilä and Folkesson et al. [9]. Histological slices from the centre of the posterior horn were stained with hematoxylin and eosin or Safranin-O-Fast Green, depending on which degenerative process was evaluated, and imaged at 40× magnification using a digital pathology slide scanner (Aperio AT2, Leica Biosystems, Wetzlar, Germany). Two observers, IK and EF, individually graded the histological images according to Pauli's histopathological scoring system [10]. The scoring system is based on degenerative changes in four categories: I. Surface integrity, with three subcategories for the femoral side, the tibial side and the inner border, II. Cellularity, III. Collagen organization and IV. Safranin-O staining intensity (relating to proteoglycan content). Each category (or subcategory) is rated from 0 to 3, resulting in a total score of 0–18, where higher values indicate a higher degree of degeneration. Between the two observers, any discrepant scores were discussed and a consensus was reached for the scores in each category. These consensus scores were then used to calculate the total score.

### 2.4 Statistical analysis

The relationship of the respective relaxation times with total Pauli score for all included menisci were evaluated using mixed effects linear models, including a random intercept to account for correlation between two samples from the same individual. The total Pauli score was treated as a continuous variable and the slope of the linear regression, with 95% confidence interval (CI), was used as a measure of association.

The relaxation times measured in two menisci from the same individual were not much more similar than relaxation times in menisci from different individuals. Therefore, the mixed effects linear models are very similar to linear models without the random intercept and thus, as a measure of model fit, we report R^2^.

The subcategories of category I reflect similar properties (surface integrity on different sides of the meniscus). Therefore, for each meniscus, a mean score was calculated for category I as a whole, to evaluate its relationship with the respective relaxation times. The mean score for category I was treated in the same way as the total score.

Categories II-IV were evaluated separately and could not be treated as continuous variables since the outcome is limited to only four discrete values. For each category, menisci were grouped based on score. In the case where there was only one meniscus with the score 0 or 3 within a particular category, they were merged into groups of 0–1 or 2–3, respectively. The associations of relaxation times with the scores for categories II-IV were analysed using mixed effects linear models with scores as ordered categorical variables, to estimate the mean difference in relaxation times for each group compared to the lowest score.

The normality and homoscedasticity of regression residuals were checked using residual plots.

## 3 Results

**Fig. 2:**
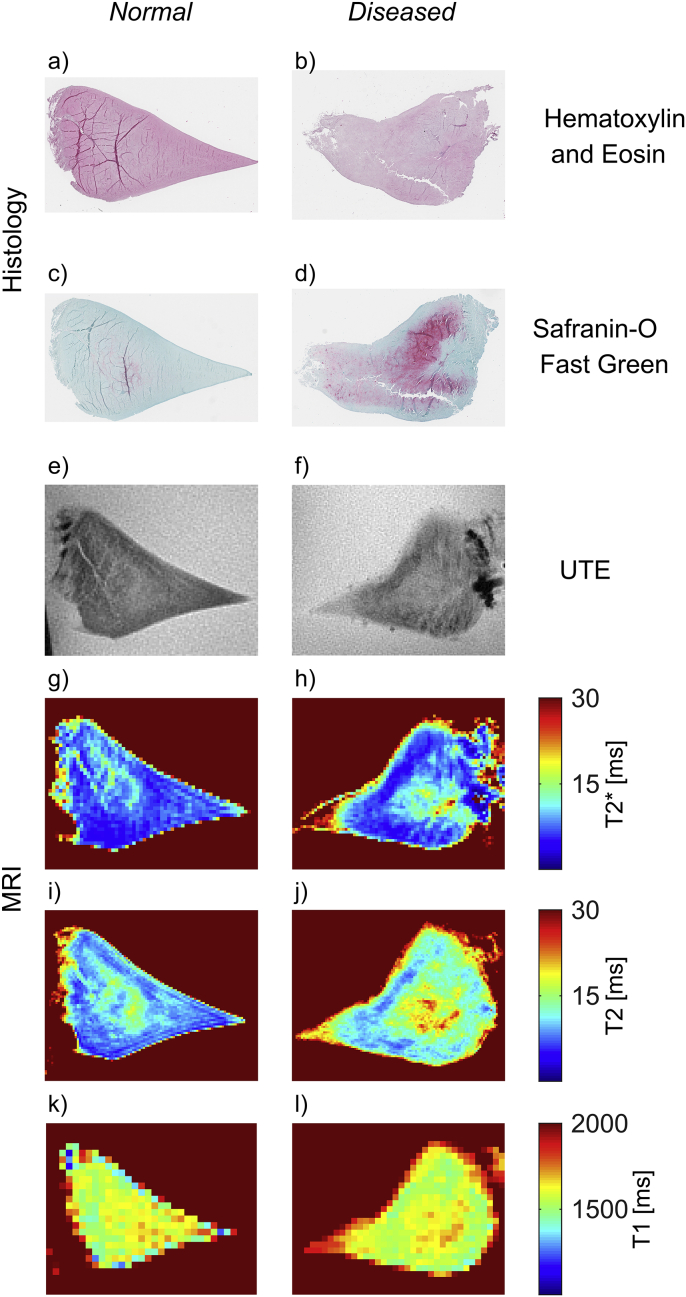
Example images of histology slices (hematoxylin and eosin staining (a–b) and Safranin-O Fast Green staining (c–d)), UTE MR images (e–f) and T2^∗^ (g–h), T2 (i–j) and T1 (k–l) maps for two menisci with total Pauli score of 4 (a, c, e, g, i and k) and 13 (b, d, f, h, j and l), respectively.

Example images of T2^∗^, T2 and T1 maps and histology slices with the two different stainings are presented for a normal (Total Pauli score 4) and a degenerated meniscus (Total Pauli score 13) in Fig. 2.

**Fig. 3:**
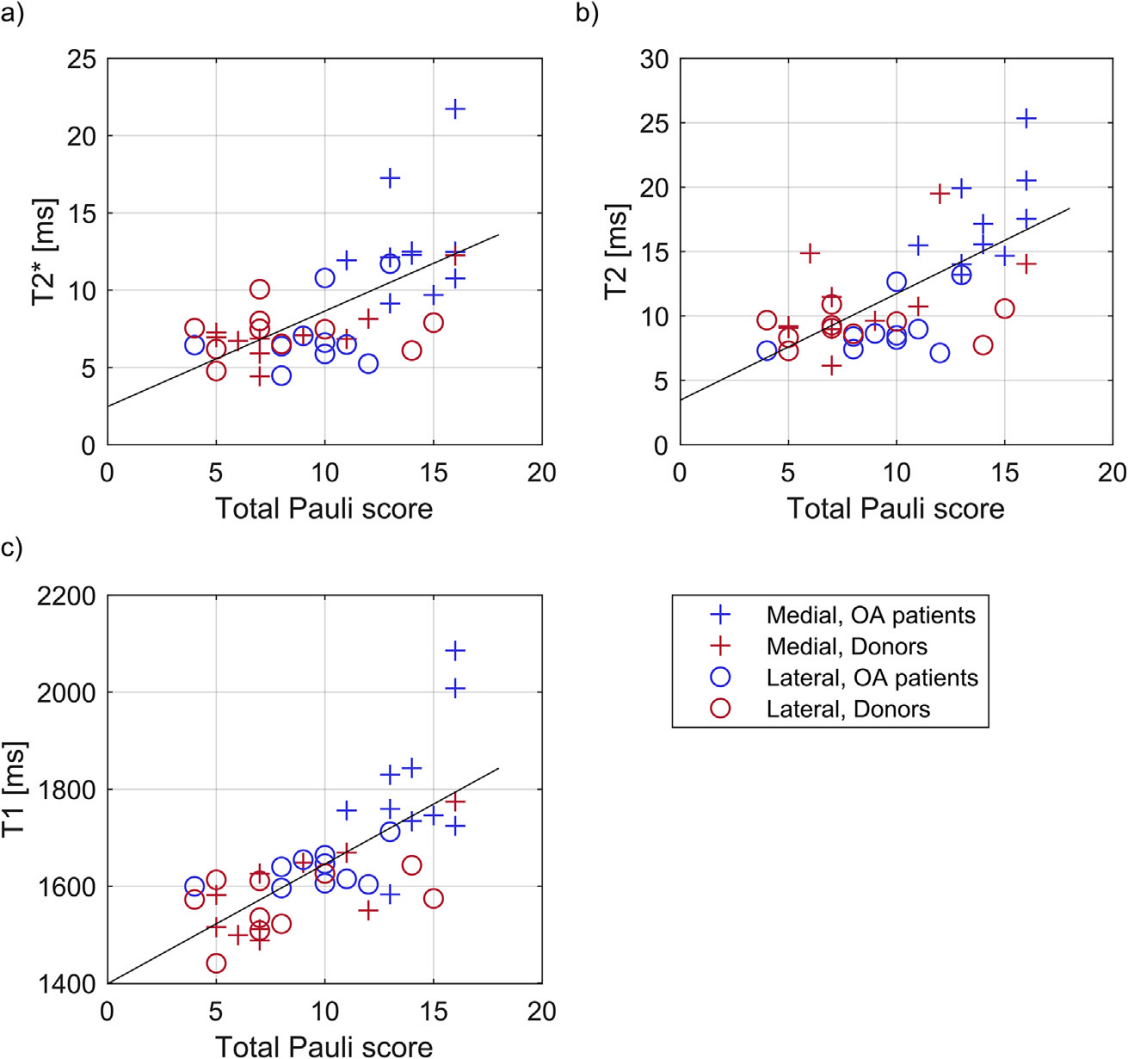
Mean relaxation times for each meniscus as a function of degeneration severity, evaluated through the total Pauli score. The lines represent the linear regression for all menisci. All three relaxation times increase with increasing degeneration.

Descriptive statistics on total Pauli score and relaxation times is presented as mean and standard deviation within each sampling group of menisci, i.e. medial and lateral menisci from OA patients and medial and lateral menisci from deceased donors, respectively (Table 2). The large variation in total Pauli score, from 4 to 16, of the included menisci indicates a large range of degenerative stages (Fig. 3).

**Table 2:** Mean values and standard deviations of total Pauli scores and relaxation times (in ms) within each meniscus group, i.e. medial and lateral menisci from OA patients and medial and lateral menisci from deceased donors without known OA.

Group		Total score		T2^∗^		T2		T1	
		Mean	SD	Mean	SD	Mean	SD	Mean	SD
OA patients	Medial	14.1	1.7	13.0	3.8	17.3	3.7	1807	146
	Lateral	9.5	2.5	7.1	2.3	9.9	2.1	1634	37
Donors	Medial	8.5	3.5	7.2	2.0	11.4	3.8	1586	92
	Lateral	8.2	3.7	7.2	1.4	9.1	1.2	1565	63

Longer T2^∗^, T2 and T1 relaxation times were measured for menisci with higher total Pauli score (Fig. 3). The increase in time per score (95% CI) was 0.62 (0.37, 0.86) ms/score for T2^∗^, 0.83 (0.53, 1.1) ms/score for T2 and 24.7 (16.5, 32.8) ms/score for T1, indicating a rather strong association between relaxation times and total Pauli score (Table 3). R^2^ for the linear regression between relaxation times and total Pauli score was, for T2^∗^ 0.38, for T2 0.43 and for T1 0.50.

**Table 3:** Mean difference, with 95% CI, in relaxation times between Pauli scores. For the total Pauli score (range 0–18) and the average score of category I (range 0–3), the difference in relaxation time is presented per score. For categories II-IV, the difference is presented between groups of different scores. The values were assessed through mixed effects linear regression for the total score and for the individual score in each category. All values are in the unit of ms.

Category		T2^∗^		T2		T1	
		Mean diff	95% CI	Mean diff	95% CI	Mean diff	95% CI
Total	per score	0.62	0.37, 0.86	0.83	0.53, 1.1	24.7	16.5, 32.8
I	per score	3.0	1.8, 4.2	4.0	2.5, 5.5	116	75.6, 156
II	0–1	1.9	−1.2, 5.0	0.90	−3.0, 4.8	90.8	−10.9, 193
	0-2/3	2.5	−0.12, 5.0	3.0	−0.22, 6.3	137	45.7, 228
III	0/1-2	1.2	−0.78, 3.2	1.5	−1.4, 4.4	43.1	−29.6, 116
	0/1-3	5.3	1.6, 8.9	6.1	1.7, 11	204	75.9, 332
IV	0–1	1.1	−3.0, 5.2	2.1	−2.7, 6.9	20.6	−123, 164
	0–2	2.9	−1.2, 6.9	4.5	−0.25, 9.3	111	−36.0, 257
	0–3	4.7	0.041, 9.5	8.2	2.6, 14	130	−35.7, 296

I: surface integrity, II: cellularity, III: collagen organization, IV: Safranin-O staining intensity.

**Fig. 4:**
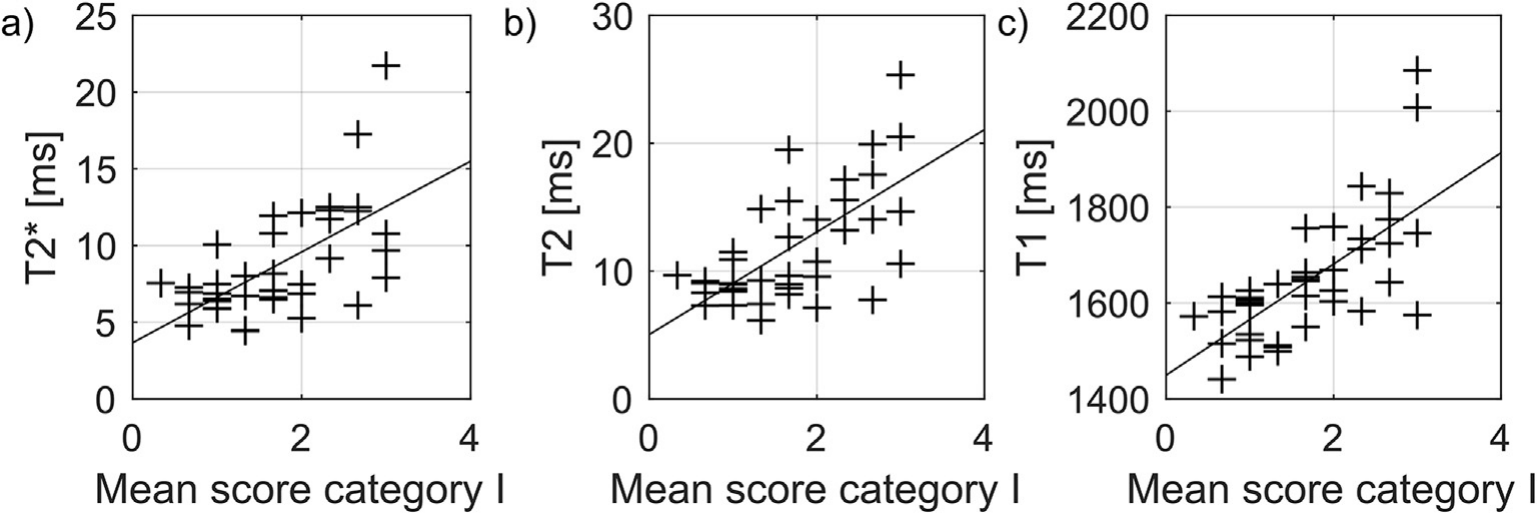
Mean relaxation times for each meniscus sample, as a function of histopathological score for category I (grading surface integrity) averaged over the sub categories for femoral, tibial and inner border surfaces. The lines represent the mixed effects linear model. Calculated regression coefficients are listed in Table 3.

**Fig. 5:**
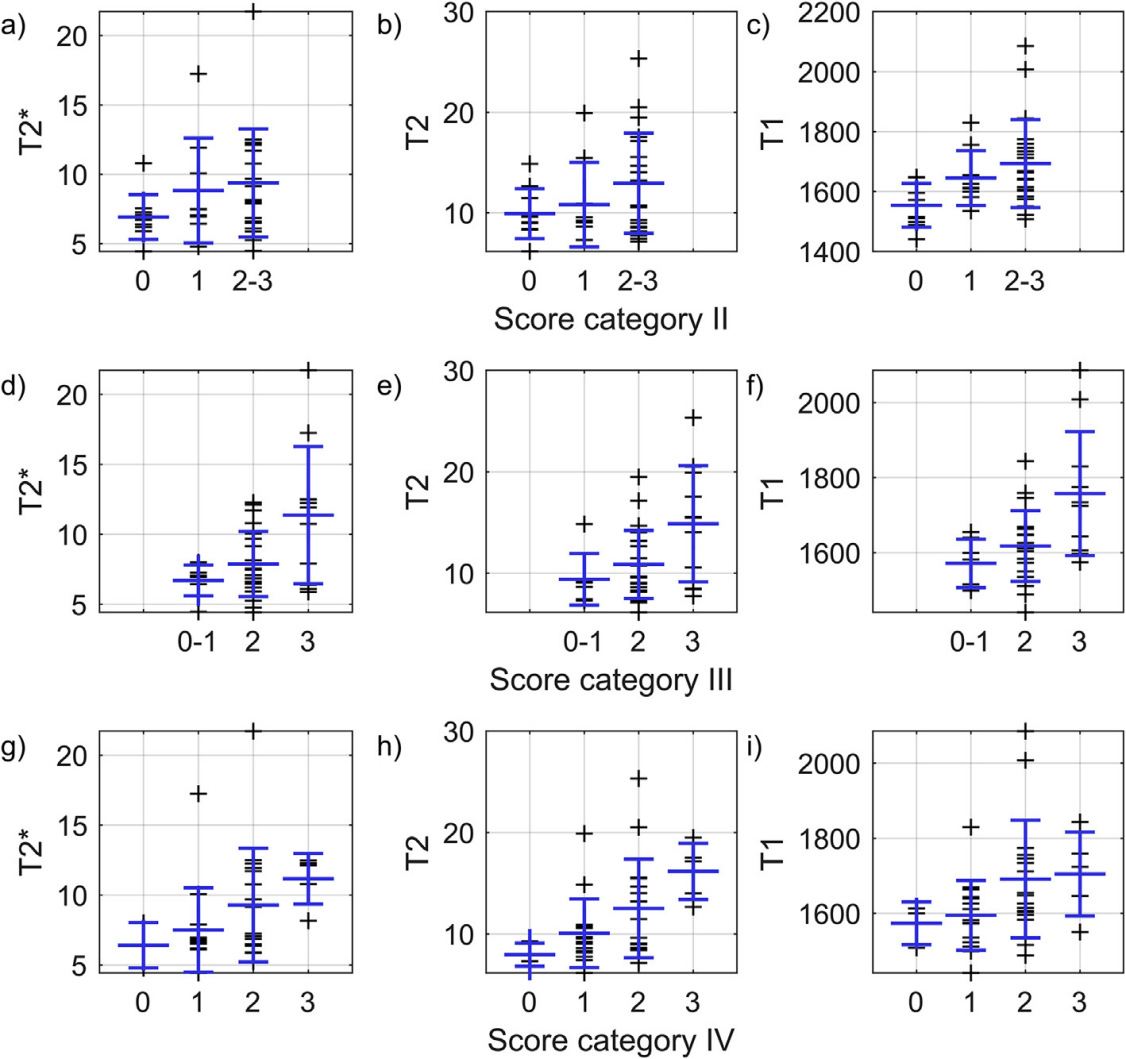
T2^∗^, T2 and T1 as a function of Pauli scores in a-c) category II (cellularity), d-f) category III (collagen organization) and g-i) category IV (Safranin-O staining intensity). Large blue markers indicate mean value and standard deviation within each group.

Further, all three relaxation parameters were associated specifically with surface integrity (Table 3, Fig. 4). Mean relaxation times generally also increased with increasing scores for categories II-IV (Table 3, Fig. 5). However, the uncertainty around the estimates was larger. Mean difference in relaxation times between groups of the lowest and highest scores, suggested all three relaxation times were associated with collagen organization (category III). An association was seen also for T1 with cellularity (category II) and for T2 with Safranin-O staining intensity (category IV).

## 4 Discussion

In this study we investigated the relationship between MR relaxation times, T2, T2^∗^ and T1, measured in the posterior horn of *ex vivo* human menisci, and degenerative processes assessed through histopathological scoring. The most important finding of our study is that all three relaxation times increased with increasing histopathological score and reflected the wide span of degenerative stages. This indicates that MR relaxation parameters may be sensitive to degeneration progression in the meniscus. The results adds to those of our previously presented study where we found that relaxation times are longer in menisci from knee compartments severely affected by OA compared to more healthy compartments [5]. The association of relaxation times with the scores for estimation of surface integrity and collagen organization could possibly be explained to some extent by an increased amount of free water in the tissue as a result of disruption of the tissue surface and the collagen network. The relationship between T1 and cellularity is less intuitive but has been reported earlier by Nebelung et al. [6]. T2 has also been suggested to reflect proteoglycan content in meniscus and articular cartilage, although previous studies investigating this relationship have yielded mixed results [[11], [12], [13], [14], [15], [16]]. For the remaining comparisons made in this study (T2^∗^ and T2 with cellularity, T2^∗^ and T1 with Safranin-O staining intensity), the mean differences and confidence intervals indicated that if an association existed, it would have been positive, i.e. relaxation times increase with increasing histopathological scores. This suggests that the increase in MR relaxation times seen with degeneration is not dependent solely on one process.

Similar to our results, Nebelung et al. reported that T1, T2 and T2^∗^ increased with tissue degeneration assessed through histopathological scoring [6]. They reported a correlation specifically for tissue integrity and cellularity. However, in contrast to our results, they did not see an association with collagen organization. The reason for this dissimilarity may be a difference in degeneration grades between the two sample materials where more samples in higher grades of degeneration could help to reveal an association.

At visual inspection, tears were found outside the evaluated area in two of the lateral donor menisci. These menisci were nevertheless included, since the purpose of including both donors and patients in this study was to ensure a variety of histopathological scores.

The preclinical 9.4 T scanner used in this study is well suited for imaging of small samples and the high field strength offers a high signal to noise ratio. Relaxation times are to some extent dependent on field strength, especially T1 that is expected to be longer at higher field strength [17]. Local field inhomogeneity may be larger at higher field strength which will then also affect T2^∗^. On the other hand, the smaller bore of a preclinical scanner compared to a human whole body scanner usually makes shimming easier and may, at least to some extent, compensate for the higher field strength. Even if the relaxation times quantified in this study cannot be directly transferred to clinical field strengths we still expect the associations with degeneration observed here to remain. The results of Eijgenraam et al. who reported an association between meniscus T2 measured *in vivo* at 3 T and total Pauli score [8], are promising for future studies *in vivo* at clinical field strengths.

The meniscus has been suggested to include both shorter and longer T2 components [[18], [19], [20]] and biexponential fitting could possibly separate them. However, it has also been reported that a monoexponential approach is sufficient for estimation of T2^∗^ in the meniscus [21], and with the relatively small number of echoes with measurable signal we collected, a monoexponential model was considered more stable.

It is not obvious which model to use when relating MR relaxation times to the histopathological scoring, and it is not certain that a linear model is the most suitable. However, in a sensitivity analysis (data not shown), we evaluated both linear and non-linear models, and since they both yielded very similar estimates for the associations we chose to only present the results from the linear models.

### 4.1 Limitations

We used thawed menisci in this study, and the freezing and thawing process could possibly contribute to further disruption of the collagen network [22] which in turn could affect for example T2 and T2^∗^ in a similar way as degeneration. However, all samples were treated in the same way and any damage caused by the freezing and thawing that would affect relaxation times would likely also be reflected in the histopathological score.

Due to the limited sample size, the number of samples with a specific score in each category is small, which limits the possibility to draw conclusions about the effects of the separate degeneration processes.

We did not assess repeatability in this study. Such evaluation is important in order to relate measurement uncertainty to effect size and would be of great interest, especially if the methods are applied in *in vivo* studies.

### 4.2 Conclusion

In conclusion, the MR relaxation times T2^∗^, T2 and T1 of *ex vivo* human meniscus posterior horns, increase with degeneration severity as assessed through histopathological scoring. Changes in these parameters seem to reflect tissue degeneration, especially related to surface integrity and collagen organization. MR relaxation times may thus be potential biomarkers for meniscus degeneration. However, further studies on the meniscus and OA development are needed to see if these results also translate to the *in vivo* case.

## Author contributions

EE: Study design, sample preparation, MRI measurements, data analysis, interpretation of results, drafting of manuscript. JS: Study design, MRI measurements, interpretation of results, manuscript revision. EF: Sample preparation, histopathological scoring, manuscript revision. IK: Histopathological scoring, manuscript revision. JT: Patient recruitment, collection of menisci, manuscript revision. PP: Study design, MRI measurements, interpretation of results, manuscript revision. MF: Histological preparation, manuscript revision. VH: Sample preparation, manuscript revision. AT: Statistical expertise, interpretation of results, manuscript revision. SS: Study design, manuscript revision. ME: Study design, sample preparation, interpretation of results, manuscript revision. All authors have approved the final version of manuscript for submission. Professor M. Englund (martin.englund@med.lu.se) takes responsibility for the integrity of this work as a whole.

## Role of the funding source

This work was supported by the 10.13039/501100000781European Research Council (ERC) under the European Union's 10.13039/501100007601Horizon 2020 research and innovation programme (grant agreement No 771121), the 10.13039/501100004359Swedish Research Council, the 10.13039/501100014034Foundation for Research in Rheumatology (FOREUM), the 10.13039/501100006075Greta and Johan Kock Foundations, the 10.13039/501100007949Swedish Rheumatism Association, the Österlund Foundation, the Governmental Funding of Clinical Research program within the National Health Service (ALF), and the 10.13039/501100006738Faculty of Medicine, Lund University, Sweden. The funders had no role in study design, data collection and analysis, decision to publish, or preparation of the manuscript.

## Declaration of Competing Interest

The authors have no conflicts of interest to declare.
